# Structure of the GH9 glucosidase/glucosaminidase from *Vibrio cholerae*

**DOI:** 10.1107/S2053230X18011019

**Published:** 2018-08-06

**Authors:** Liang Wu, Gideon J. Davies

**Affiliations:** a York Structural Biology Laboratory, Department of Chemistry, The University of York, York YO10 5DD, England

**Keywords:** glycoside hydrolase, enzymes, carbohydrates, VC0615, glucosidase, glucosaminidases, *Vibrio*, hydrolases

## Abstract

Glycoside hydrolase family 9 (GH9) of carbohydrate-processing enzymes primarily consists of inverting endoglucanases. A subgroup of GH9 enzymes are believed to act as exo-glucosidases or exo-glucosaminidases, with many being found in organisms of the family Vibrionaceae, where they are proposed to function within the chitin-catabolism pathway. Here, it is shown that the GH9 enzyme from the pathogen *Vibrio cholerae* (hereafter referred to as VC0615) is active on both chitosan-derived and β-glucoside substrates. The structure of VC0615 at 3.17 Å resolution is reported from a crystal form with poor diffraction and lattice disorder. VC0615 was highly refractory to crystallization efforts, with crystals only appearing using a high protein concentration under conditions containing the precipitant poly-γ-glutamic acid (PGA). The structure is highly mobile within the crystal lattice, which is likely to reflect steric clashes between symmetry molecules which destabilize crystal packing. The overall tertiary structure of VC0615 is well resolved even at 3.17 Å resolution, which has allowed the structural basis for the exo-glucosidase/glucosaminidase activity of this enzyme to be investigated.

## Abstract 2

The structure of a *Vibrio cholerae* ‘exo’-glycosidase from CAZy family GH9 has been solved at 3.17 Å resolution. Preliminary activity assays show that the enzyme is active towards both chitosan-derived oligosaccharides and β-glucosides.

## 1. Introduction   

*Vibrio cholerae* is the pathogen responsible for cholera, a disease characterized by severe diarrhoea that is estimated to affect ∼3 million people worldwide and causes nearly 100 000 deaths per annum (Ali *et al.*, 2015 ▸). Bacteria of the family Vibrionaceae, which includes the *Vibrio* genus, occur naturally as members of the marine bacterioplankton community, where they form part of the bacterial flora of chitinous zooplankton. Vibrionaceae readily utilize chitin as a source of organic carbon and nitrogen, and their attachment to chitinous zooplankton such as copepods has been hypothesized to provide a nutrient-rich habitat for the bacteria (Heidelberg *et al.*, 2002 ▸). The association of *V. cholerae* with copepods contributes significantly to the cholera disease burden, such that an increased copepod number in water sources is positively correlated with increased outbreaks of cholera (Huq *et al.*, 2005 ▸). Chitin has also been suggested to directly affect the physiology of *V. cholerae*, for example by protecting the bacteria from cold stress (Amako *et al.*, 1987 ▸) or from killing by gastric acid (Nalin *et al.*, 1979 ▸). The key importance of chitin in the life cycle of *V. cholerae* and other Vibrionaceae demands a detailed understanding of the biochemical pathways responsible for chitin utilization by these organisms, many of which contribute significantly to the global disease burden.

The *V. cholerae* enzyme VC0615 was initially identified as a putative inverting exoglucosidase (termed BglA) based on its clear exo activity on cello-oligosaccharides and aryl-β-glucosides and its apparent lack of activity on β-1,4-linked di­saccharides of GlcN and GlcNAc (Park *et al.*, 2002 ▸). Based on sequence homology, VC0615 was assigned to glycoside hydrolase family 9 (GH9) of the Carbohydrate-Active enZymes (CAZy) classification scheme (Cantarel *et al.*, 2009 ▸; The CAZypedia Consortium, 2018 ▸), a family primarily associated with endoglucanases (‘cellulases’). Given that VC0615 was found in a chitin-utilization operon, this apparent inconsistency between specificity and operon perplexed the authors, as they understood that *V. cholerae* was unable to metabolize cellobiose or other higher cello-oligosaccharides.

In 2008, a large-scale assessment of chitin-utilization pathways in the Vibrionaceae suggested that VC0615 was in fact a exo-acting glucosaminidase, the substrate of which was the GlcN-GlcN-O6-P disaccharide resulting from the import of chitobiose (GlcN)_2_ through a phosphotransferase-transport system (Hunt *et al.*, 2008 ▸). [Note: here we use chitobiose to refer to the β-1,4 (GlcN)_2_ disaccharide and *N*,*N*′-diacetyl­chitobiose to refer to the β-1,4 (GlcNAc)_2_ disaccharide, as outlined in the IUPAC–IUB naming convention guidelines (Nomenclature Committee of IUB and IUPAC–IUB Joint Commission on Biochemical Nomenclature, 1988 ▸).]

More recently, Honda and coworkers showed that a close (60% identity) homologue of VC0615, PBPRA0520 from *Photobacterium profundum* SS9, was indeed a multifunctional exo-acting glucosaminidase/β-glucosidase, with a *k*_cat_/*K*_m_ at least ten times greater for chito-oligosaccharides compared with the corresponding cello-oligosaccharides, supporting the functional assignment of this enzyme as an exo-acting glucos­aminidase active on chitobiose substrates (Honda *et al.*, 2011 ▸). Subsequently, a crystal structure of PBPRA0520 confirmed this enzyme to be an exo-acting GH9, illustrating that loop extensions (compared with endo-acting GH9 enzymes) blocked the −4 to −2 subsites of the enzyme, rendering it unable to bind substrates in an endo fashion (Honda *et al.*, 2016 ▸).

To date, 16 different three-dimensional structures are known for CAZy family GH9. 13 of these known structures are endoglucanases, reflecting their dominance within the GH9 family. One structure of a *Clostridium thermocellum* cellobiohydrolase (CbhA) has also been reported. Whilst not strictly an exo-acting enzyme, as it hydrolyses terminal cellobiose units rather than terminal glucose units, Schubot and coworkers classified CbhA as an ‘exocellulase’ based on the closed topology of the active site of the enzyme, which obstructs the binding of substrates beyond the −2 subsite (Schubot *et al.*, 2004 ▸; Davies *et al.*, 1997 ▸). PBPRA0520 and a closely related enzyme from *V. parahaemolyticus* (VP2484) are the only exo-acting GH9 enzymes with solved three-dimensional structures, although VP2484 has yet to be fully biochemically characterized.

Here, we report the structure solution of the *V. cholerae* enzyme VC0615, which was solved at 3.17 Å resolution with poor-quality data reflecting crystal lattice disorder. In contrast to previous analyses (Park *et al.*, 2002 ▸), we find that VC0615 is substantially more active on (GlcN)_2_ than (Glc)_2_, although the hydrolysis of glucosides by VC0615 was still sufficient to determine Michaelis–Menten kinetic parameters for aryl β-glucoside substrates. Despite the inferior data, the 3.17 Å resolution structure was sufficient to confirm the structural basis of how this enzyme functions as an exo-glycosidase through extended loops from α-helices 1–2 and α-helices 9–10, which block substrate binding beyond the −1 subsite.

## 2. Materials and methods   

### 2.1. Macromolecule production   

A synthesized cDNA encoding VC0615, cloned into the NdeI (5′) and XhoI (3′) sites of the pET-21a vector, was purchased from GenScript. This construct contains the full VC0615 gene followed by a C-terminal hexahistidine tag. The correctness of the cloned plasmid was verified by sequencing with the T7-fwd and pET-RP primers (Table 1 ▸) prior to use in protein production.

The pET-21a-VC0615 plasmid was used to transform chemically competent *Escherichia coli* BL21 Gold (DE3) cells. Cells were grown for protein production in Terrific Broth (TB) containing ampicillin (100 µg ml^−1^) at 37°C with shaking. Upon reaching an optical density at 600 nm (OD_600_) of 0.8, the cultures were induced with 0.5 m*M* isopropyl β-d-1-thio­­galactopyranoside (IPTG), followed by incubation at 16°C overnight with shaking.

The cells were harvested by centrifugation at 4000*g* for 15 min at 4°C. The pellet was resuspended in HisTrap buffer *A* (20 m*M* Tris pH 8.0, 500 m*M* NaCl, 20 m*M* imidazole, 1 m*M* DTT) supplemented with EDTA-free cOmplete protease-inhibitor cocktail (Roche) and bovine pancreatic DNase I (Sigma), and the cells were lysed by passage through a cell disruptor (Constant Systems) at a pressure of 172 MPa. The cell lysate was cleared by centrifugation at 50 000*g* for 45 min at 4°C and the cleared lysate was immediately applied onto a 5 ml HisTrap FF crude column (GE Healthcare) pre-equilibrated with HisTrap buffer *A*. Following the application of the lysate, the column was washed with five column volumes (CV) of HisTrap buffer *A* to remove unbound protein, followed by elution of the bound protein using an increasing linear gradient of HisTrap buffer *B* (20 m*M* Tris pH 8.0, 500 m*M* NaCl, 1000 m*M* imidazole, 1 m*M *DTT) over 20 CV. SDS–PAGE was used to identify fractions containing VC0615, which were pooled and concentrated to a volume of ∼5 ml using a 30 kDa cutoff Vivaspin centrifugal concentrator (Millipore). This concentrated protein from HisTrap purification was purified by size-exclusion chromatography (SEC) using a S75 16/600 column (GE Healthcare) pre-equilibrated with SEC buffer (20 m*M* HEPES pH 7.4, 200 m*M* NaCl, 1 m*M* DTT). SEC fractions containing VC0615 were identified by SDS–PAGE, pooled and diluted 20-fold in ion-exchange (IEX) buffer *A* (20 m*M* HEPES pH 7.4, 1 m*M* DTT). The diluted protein sample was applied onto a 5 ml HiTrap Q HP column (GE Healthcare) pre-equilibrated in IEX buffer *A*. The loaded column was washed with 5 CV IEX buffer *A* and eluted with a 20 CV increasing linear gradient of IEX buffer *B* (20 m*M* HEPES pH 7.4, 1500 m*M* NaCl, 1 m*M* DTT). SDS–PAGE identified the main IEX elution peak to contain pure VC0615, whilst a small shoulder peak eluting at higher NaCl concentrations contained contaminant proteins.

Fractions containing purified VC0615 were pooled and diluted with IEX buffer *A* to adjust the NaCl concentration to 100 m*M*. Purified protein samples were concentrated using a 30 kDa cutoff Vivaspin centrifugal concentrator and the final concentrations were calculated by UV absorbance at 280 nm (*A*_280_) using a calculated molar extinction coefficient of 129 720 *M* ^−1^ cm^−1^ and a calculated molecular mass of 66 130.81 g mol^−1^.

### 2.2. Kinetic assays   

Enzyme kinetics were determined by measuring the hydrolysis of the artificial fluorogenic substrate 4-methyl­umbelliferyl-β-d-glucopyranoside (4MU-β-d-Glc; Sigma). In brief, 50 µl solutions of 4MU-β-d-Glc at 2× final concentration in 20 m*M* sodium phosphate pH 6.5 were prepared in a 96-well microplate (Nunc). To initiate the reaction, 50 µl of VC0615 at a concentration of 2 µ*M* in 20 m*M* sodium phosphate pH 6.5 was added to each substrate solution, giving final reaction volumes of 100 µl containing 1× 4MU-β-d-Glc substrate and 1 µ*M* VC0615. Reactions were monitored continuously for release of the fluorescent 4MU product over 600 s using a Polarstar microplate reader (BMG Labtech). The initial reaction rates plotted against the 4MU-β-d-Glc substrate concentration were fitted by nonlinear regression to the Michaelis–Menten equation *V* = (*V*_max_[4MU-β-d-Glc])/(*K*_m_ + [4MU-β-d-Glc]). *k*_cat_ was determined from *V*_max_ through the relationship *V*_max_ = *k*_cat_[E]. All reactions were carried out in triplicate.

Reactions using other 4MU glycosides were carried out as above.

### 2.3. Thin-layer chromatography (TLC) analyses   

All oligosaccharide substrates and standards for digests and TLC analyses were purchased from Megazyme, except for (GlcN)_2_, which was purchased from Sigma. Digest reactions were carried out in McIlvaine phosphate–citrate buffer pH 6.5 using an oligosaccharide substrate concentration of 10 mg ml^−1^ and 0.5 µ*M* VC0615. 20 µl reaction volumes containing enzyme and substrate were incubated at 37°C with shaking. At set time points, 2 µl of the reaction mixture was removed and ‘paused’ by flash-freezing in liquid N_2_. Following completion of the reaction time course, all frozen aliquots were thawed and spotted onto an aluminium foil-backed silica TLC plate (Sigma).

The TLC plate was run in a 50:25:25 *n*-butanol:water:acetic acid solvent system until the solvent front reached ∼1–2 cm from the top of the plate. In order to improve the separation between spots, the TLC plate was dried and rerun a second time in the same solvent system. Following the second run, the TLC plate was dried and developed using a *p*-anisaldehyde (Sigma) stain (3.7 ml *p*-anisaldehyde, 1.5 ml acetic acid, 5 ml concentrated sulfuric acid, 135 ml ethanol) with mild heating.

### 2.4. SEC-MALLS   

SEC-MALLS experiments were run in 20 m*M* HEPES 7.4, 200 m*M* NaCl buffer. The injected sample comprised 100 µl VC0615 at 2.5 mg ml^−1^ in 20 m*M* HEPES pH 7.4, 100 m*M* NaCl, 1 m*M* DTT. Experiments were conducted on a system comprising a Wyatt HELEOS II multi-angle light-scattering detector and a Wyatt rEX refractive-index detector linked to a Shimadzu HPLC system (SPD-20A UV detector, LC20-AD isocratic pump system, DGU-20A3 degasser and SIL-20A autosampler). Work was conducted at room temperature (20 ± 2°C). All solvents and buffers were 0.2 µm filtered before use and a further 0.1 µm filter was present in the flow path. The Shimadzu *LC Solutions* software was used to control the HPLC and *ASTRA V* software was used for the HELEOS II and rEX detectors.

All data were analysed using the *ASTRA V* software. Molecular masses were estimated using the Zimm fit method with a degree of 1. A value of 0.18 ml g^−1^ was used for the protein refractive-index increment (d*n*/d*c*).

### 2.5. Crystallization   

Crystallization trials were carried out in sitting-drop vapour-diffusion format using 96-well Swissci SD-2 plates (‘MRC’ plates) against a range of commercial screens, with drops consisting of 120 nl protein and 120 nl reservoir solution. Initial trials with PEG- and/or salt-based crystallization screens using an ∼30 mg ml^−1^ stock of VC0615 protein did not yield any hits. Subsequently, the protein concentration was increased to 67.5 mg ml^−1^ and a further range of commercial screens were tested. Crystals were found in the PGA screen (Molecular Dimensions; Hu *et al.*, 2008 ▸) under conditions containing 0.1 *M* Tris buffer pH 7.8 and a combination of PEG and PGA-LM precipitants. Optimization yielded crystals at basic pH values across a range of PEG and PGA-LM concentrations in both sitting-drop and hanging-drop vapour-diffusion formats. The final crystallization conditions were 0.1 *M* Tris pH 7.5, 5% PGA-LM, 8% PEG 20K in a hanging-drop vapour-diffusion format using 1 µl protein solution and 1 µl reservoir solution.

Substantial difficulty with cryoprotection was experienced during the crystal-harvesting process. Harvesting crystals into a ‘standard’ cryoprotectant consisting of the crystallization well solution (0.1 *M* Tris pH 7.5, 5% PGA-LM, 8% PEG 20K) supplemented with 25% ethylene glycol resulted in the crystals rapidly dissolving. Extensive experimentation found that the stable cryoprotection of crystals necessitated the use of high concentrations of PGA-LM, although the removal of PEG 20K did not adversely affect the stability or diffraction of the crystals. Thus, crystals were harvested into an optimized cryoprotectant solution consisting of 0.1 *M* Tris pH 7.5, 13% PGA-LM, 25% ethylene glycol before flash-cooling in liquid nitrogen for data collection. Crystallization information is summarized in Table 2 ▸.

### 2.6. Data collection and processing   

Data were collected on beamline I03 at Diamond Light Source (DLS), UK. Images were indexed and integrated with *XDS* (Kabsch, 2010 ▸), followed by data reduction and scaling with *AIMLESS* (Evans & Murshudov, 2013 ▸). All calculations were carried out within the new *CCP*4*i*2 interface to the *CCP*4 software suite (Potterton *et al.*, 2018 ▸). Data-collection statistics are summarized in Table 3 ▸.

### 2.7. Structure solution and refinement   

The crystal structure of VC0615 was solved by molecular replacement (MR) using both *MOLREP* (Vagin & Teplyakov, 2010 ▸) and *Phaser* (McCoy, 2007 ▸) with a monomer of the GH9 enzyme from *V. parahaemolyticus* (PDB entry 3h7l; New York SGX Research Center for Structural Genomics, unpublished work) as the search model in each case. The top MR solution from *MOLREP* found four chains in the asymmetric unit, whilst the top MR solution from *Phaser* found five chains. Closer inspection revealed that electron density for one chain in the *Phaser* solution was substantially poorer than for the others, suggesting either disorder or partial occupancy. It was decided to leave this chain in the model, as positive *F*_c_ − *F*_o_ difference density and higher *R*_work_ and *R*_free_ factors were observed in its absence.

Because *MOLREP* incorporates alignment and modification of the search model against the target sequence, MR using *Phaser* was performed again using a monomer that had been aligned and modified by *MOLREP*. The resulting solution contained five chains in the asymmetric unit, each with the correct VC0615 sequence. The molecular-replacement solution from *Phaser* was subjected to alternating rounds of manual model building in *Coot* (Emsley & Cowtan, 2004 ▸) and refinement with *REFMAC*5 (Murshudov *et al.*, 2011 ▸) using automatically generated local NCS restraints. The refinement statistics are summarized in Table 4 ▸. Quaternary-structure assemblies were analysed using *PISA* (Krissinel & Henrick, 2007 ▸) and dimer coordinates were generated using the *PISA* extension within *Coot*. Root-mean-square deviations (r.m.s.d.) were calculated using the SSM superpose function of *CCP*4*mg* (McNicholas *et al.*, 2011 ▸). Crystallographic figures were generated using *PyMOL* (Schrödinger).

## 3. Results and discussion   

### 3.1. Biochemical characterization of recombinant VC0615   

**Figure 1:**
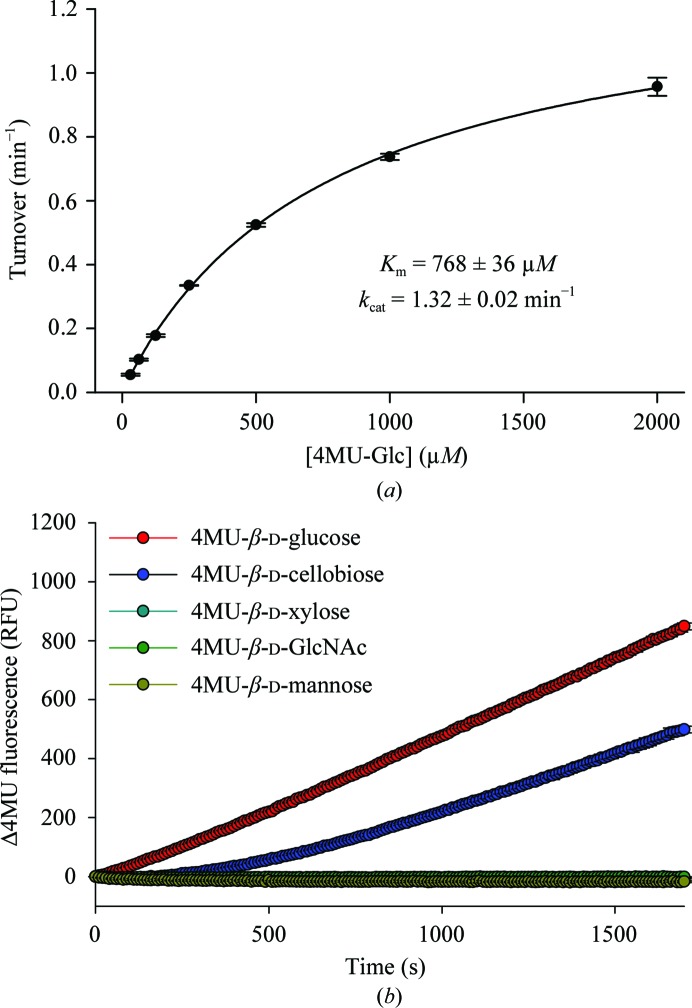
Hydrolysis of fluorogenic substrates by VC0615. (*a*) Michaelis–Menten kinetics for the hydrolysis of 4MU-β-d-glucose by VC0615. Error bars are standard deviations from three technical replicates. (*b*) Hydrolytic release of 4MU from various substrates. Only 4MU-β-d-glucose and 4MU-β-d-cellobiose show any hydrolysis. The release of 4MU from 4MU-β-d-cellobiose follows a lag period, consistent with processive hydrolysis by an exo-acting enzyme. (RFU, relative fluorescence units.)

We first characterized the enzymatic activity of purified recombinant VC0615 using the fluorogenic substrates 4MU-β-­d-glucose and 4MU-β-d-cellobiose. In line with previous analyses of this enzyme (Park *et al.*, 2002 ▸), 4MU-β-d-glucose was hydrolysed by VC0615, with Michaelis–Menten kinetic parameters *K*_m_ = 768 µ*M* and *k*_cat_ = 1.3 min^−1^ (Fig. 1 ▸ *a*). Consistent with its reported activity as an exo-acting enzyme, we were unable to model Michaelis–Menten kinetics for the hydrolysis of 4MU-β-d-cellobiose by VC0615, as the release of 4MU was preceded by a lag period reflecting prior hydrolysis of a −2 glucose before the release of 4MU. No hydrolysis was observed against 4MU-β-d-xylose, 4MU-β-d-GlcNAc or 4MU-β-d-mannose substrates (Fig. 1 ▸ *b*). Honda and coworkers have previously shown PBPRA0520 from *P. profundum* (a close homologue of VC0615) to be a competent exo-glucosaminidase (Honda *et al.*, 2011 ▸). However, we were unable to measure kinetic parameters for the hydrolysis of 4MU-β-d-glucosamine by VC0615 owing to a lack of commercial availability of this substrate.

**Figure 2:**
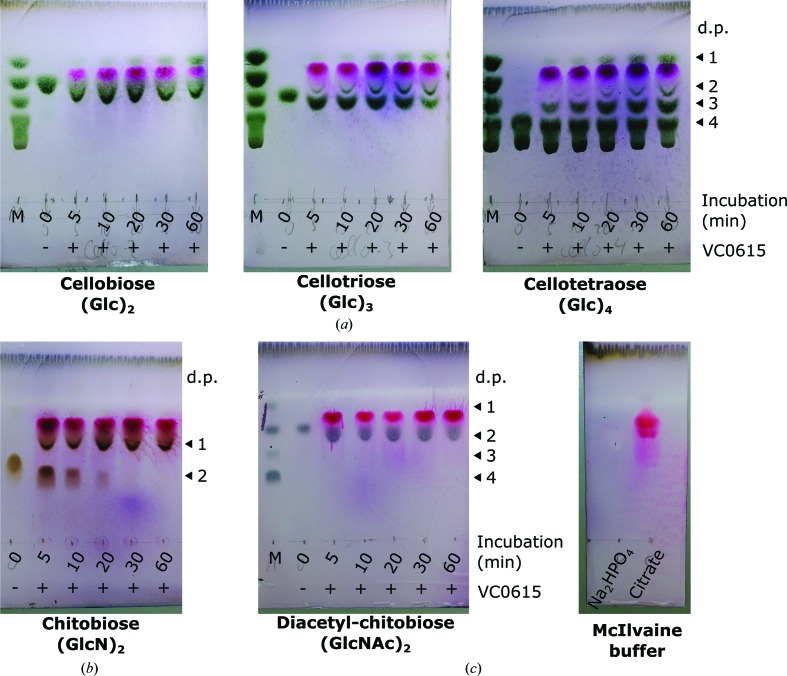
TLC analysis of cello-oligosaccharide and chito-oligosaccharide substrate hydrolysis by VC0615. (*a*) VC0615 digestion of cello-oligosaccharides effects the gradual release of glucose and shorter oligosaccharides, consistent with the activity of a processive exo-glycosidase. (*b*) VC0615 effects the complete digestion of chitobiose within 30 min, compared with the relatively slow hydrolysis observed for the cello-oligosaccharides. (*c*) VC0615 does not hydrolyse *N*,*N*′-diacetylchitobiose. In all TLCs, the purple spot corresponds to citrate from the McIlvaine reaction buffer, which was not present in the standards. The citrate causes some displacement of lower spots. (d.p., degree of polymerization.)

In order to obtain a semi-quantitative measure of the efficiency of glucosidase *versus* glucosaminidase activity by VC0615, we next investigated the hydrolysis of various oligosaccharides by VC0615 using thin-layer chromatography (TLC). When cellobiose, cellotriose or cellotetraose were used as substrates for VC0615, we observed a gradual increase in glucose monomers and shorter cello-oligosaccharides in the reaction mixture. The cleavage pattern observed was consistent with the processive removal of single glucose units from cello-oligosaccharide chains, rather than internal chain hydrolysis by an endo-glycosidase (Fig. 2 ▸ *a*).

When chitobiose (GlcN)_2_ was used as the substrate for VC0615, cleavage to the monosaccharide was also observed (Fig. 2 ▸ *b*). Strikingly, a large amount of product was already visible after 5 min reaction time and VC0615 effected a complete digestion of the chitobiose substrate within 30 min. In contrast, a substantial amount of starting material remained in the cellobiose digest after 1 h (both reactions were initiated with 10 mg ml^−1^ substrate). These experiments demonstrate that VC0615 is a substantially more effective exo-glucos­aminidase than exo-glucosidase, which is in line with previous observations on PBPRA0520 (Honda *et al.*, 2011 ▸) and the lack of cellulose metabolism shown by Vibrionaceae in general. Consistent with the 4MU substrate experiments, no digestion of *N*,*N*′-diacetylchitobiose (GlcNAc)_2_ was observed even after 1 h incubation (Fig. 2 ▸ *c*).

### 3.2. Crystallization and crystal-packing interactions of VC0615   

**Figure 3:**
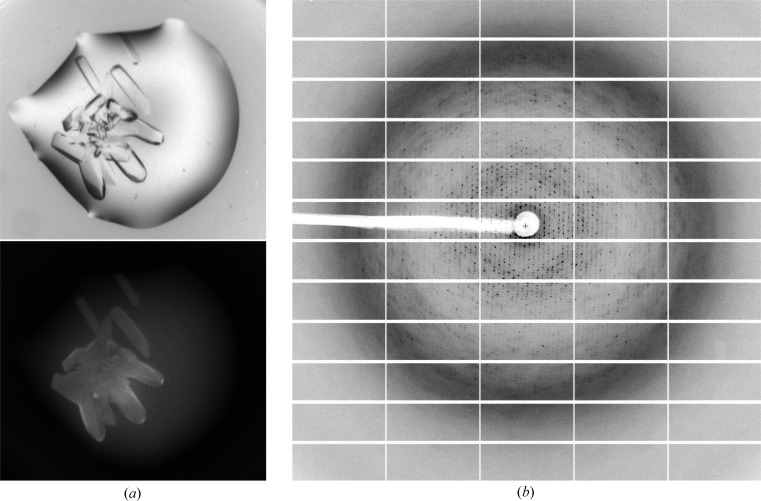
Crystallization and diffraction of VC0615. (*a*) Bright-field and UV fluorescence images of VC0615 crystals from PGA screen well G8 (0.1 *M* Tris pH 7.8, 5% PGA-LM, 8% PEG 20K). (*b*) Diffraction image of a VC0615 crystal on beamline I03 at Diamond Light Source.

A first round of crystallization trials of VC0615 were carried out at ∼30 mg ml^−1^ protein concentration using a range of PEG and/or salt precipitant screens. We found no protein crystals in any of the conditions tested. Subsequently, the VC0615 concentration was increased to ∼67.5 mg ml^−1^ and additional crystallization screens were tested. In this second round of screening we observed several hits in the PGA screen (Molecular Dimensions) under conditions containing Tris buffer and a combination of PGA-LM and PEG precipitants (Fig. 3 ▸ *a*). Optimization trials indicated that crystallization favoured basic pH values, with a range of PGA-LM and PEG concentrations being tolerated. Both sitting-drop and hanging-drop vapour-diffusion techniques yielded crystals of similar visual quality. The final crystallization conditions were 0.1 *M* Tris pH 7.5, 5% PGA-LM, 8% PEG 20K. Crystals typically appeared within several hours and reached maximum size within 2 d (Table 2 ▸).

Despite the apparent visual quality of the optimized VC0615 crystals, it became apparent that their suitability for X-ray diffraction studies was limited. Even after extensive optimization of the crystallization and cryoprotectant conditions, all crystals of VC0615 tested showed highly smeared diffraction patterns that were indicative of substantial internal disorder within the crystal. The most promising crystals of VC0615, cryoprotected using 13% PGA-LM and 25% ethylene glycol, were sent to beamline I03 at Diamond Light Source, UK for data collection. The synchrotron diffraction images were similar in appearance to those collected in-house (Fig. 3 ▸ *b*), although the mosaicity of the data was somewhat lower than expected (0.26° for the best data set). The best data set for VC0615 was processed to 3.17 Å resolution and was used for all further calculations (Tables 3 ▸ and 4 ▸).

The diffraction of the VC0615 crystals indicated a trigonal space group with a single screw axis along the *a* axis, matching either *P*3_1_21 or *P*3_2_21. Matthews coefficient analysis suggested six or seven molecules of protein per asymmetric unit to be the most likely composition of the VC0615 crystals (with 30 and 40% probability and 53.3 and 45.5% solvent content, respectively), although five or eight molecules per asymmetric unit were also plausible (with 10 and 15% probability and 61.1 and 37.7% solvent content, respectively).

**Figure 4:**
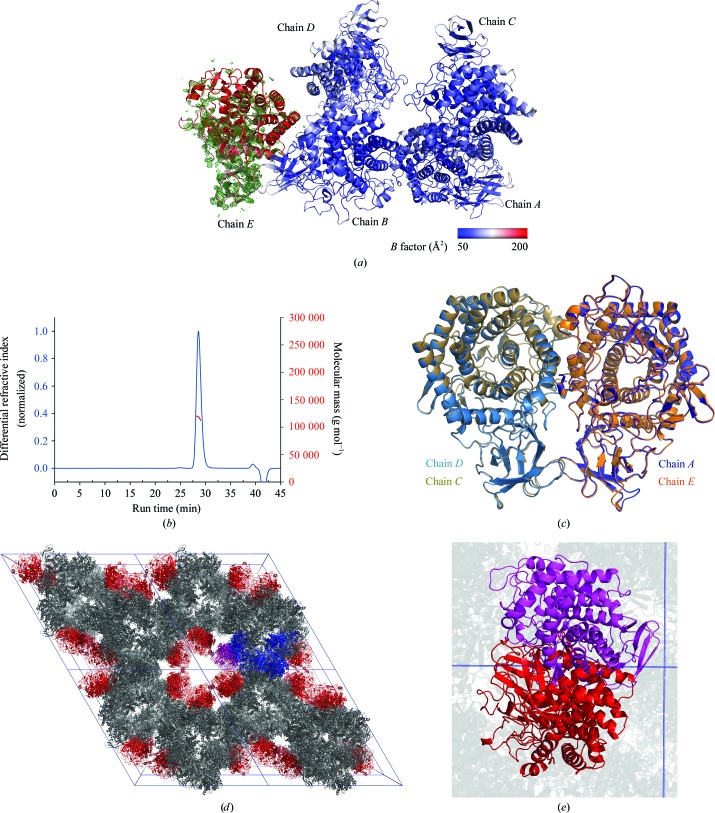
Subunit interactions and crystal packing of VC0615. (*a*) Asymmetric unit of VC0615 coloured by *B* factor, showing the substantially higher disorder of chain *E*. The map shown is *F*_c_ − *F*_o_ OMIT density contoured at 0.22 e Å^−3^ calculated from refinement of VC0615 without chain *E*. (*b*) SEC-MALLS trace showing a single peak for purified VC0615, containing a species with an estimated molecular mass of ∼118 kDa, indicating that VC0615 forms a dimer in solution. (*c*) Superposition of chains *A* and *D* and chains *B* and *C* dimers of VC0615. (*d*) Packing of VC0615 crystals. A single asymmetric unit is highlighted in blue (chains *A*–*D*) and purple (chain *E*). Chains *A*–*D* and chain *E* in other asymmetric units are coloured grey and red, respectively. A central channel surrounded by molecules of chain *E* can be observed at the intersection of four unit cells. (*e*) Steric clashes which arise between two symmetry-related molecules of a hypothetical ‘chain *F*’ (red and purple) in the VC0615 crystal lattice.

The VC0615 structure was solved by molecular replacement using the GH9 enzyme VP2484 from *V. parahaemolyticus*, which shares 68% identity with VC0615. The best molecular-replacement solution from *Phaser* indicated the correct space group to be *P*3_2_21, with five molecules of VC0615 in the asymmetric unit. Surprisingly, when the same molecular replacement was conducted with *MOLREP* using default settings, only four molecules of VC0615 were placed. Closer inspection revealed that whilst electron density for four of the five chains of VC0615 was clear and well defined, the density for the fifth chain (hereafter referred to as chain *E*) was substantially poorer, bordering on uninterpretable in many regions. Consistent with this poorer electron density, the *B* factors for chain *E* were substantially higher than for the other four chains (hereafter referred to as chains *A*–*D*) (Fig. 4 ▸ *a*).

To establish the degree of oligomerization for VC0615 in solution, we analysed purified protein by size-exclusion chromatography coupled to multi-angle laser light scattering (SEC-MALLS). Purified VC0615 eluted as a single major peak with a calculated molecular mass of ∼118 kDa, indicating that the species in solution was likely to be a dimer (monomer mass of ∼66 kDa; Fig. 4 ▸ *b*). *PISA* analysis (Krissinel & Henrick, 2007 ▸) suggested stable dimers between molecules of chain *A* and chain *D* of an adjacent asymmetric unit, and also between chain *B* and chain *C* of an adjacent asymmetric unit. Calculated interface areas were 1294.9 Å^2^ per molecule for the *A*–*D* dimer and 1289.6 Å^2^ per molecule for the *B*–*C* dimer. Given the essentially identical calculated interface areas between the *A*–*D* and *B*–*C* dimers, as well as their strong similarity (r.m.s.d. of 0.32 Å over 1129 residues as calculated by SSM superposition in *CCP*4*mg*), this arrangement of VC0615 is likely to be the biologically relevant dimer present in solution (Fig. 4 ▸c).

Analysis of protein-packing interactions within the VC0615 crystal lattice may provide some explanation for the substantially poorer electron density observed for chain *E*. The crystals of VC0615 appeared to contain a large central channel parallel to the *c* axis at the intersection of four adjacent unit cells, which is bordered by molecules of chain *E* (Fig. 4 ▸ *d*). Given the dimeric nature of VC0615, we speculated on the possibility of a sixth ‘chain *F*’ of VC0615 *in crystallo*, which might lie within this central channel as the dimer partner of chain *E*. Although some positive *F*_c_ − *F*_o_ difference density was observed in the central channel, this density was too poor to model even a small portion of VC0615. Thus, we examined the effect of adding ‘chain *F*’ to the VC0615 crystal structure by modelling its predicted position through the superposition of the *A*–*D* dimer onto chain *E*. The resulting hypothetical six-chain model revealed a large steric clash between ‘chain *F*’ and a symmetry equivalent along the *c* axis, demonstrating that an additional molecule of VC0615 cannot be readily accommodated in this crystal-packing arrangement (Fig. 4 ▸ *e*). Solving the structure of VC0615 in the lower symmetry space group *P*3_2_ did not produce a structure in which the ‘chain *F*’ molecules were well resolved, indicating that the observed symmetry clash was unlikely to result from a pseudosymmetry operator being mistaken for crystallographic symmetry in the *P*3_2_21 structure. Given that ‘chain *F*’ is the dimer partner of chain *E*, difficulty in packing this chain readily explains the disorder observed for molecules of chain *E* within the VC0615 crystal structure.

When the VC0615 structure was refined with only chains *A*–*D* in the asymmetric unit, the *R*_work_ and *R*_free_ factors were ∼2–3% higher and substantial positive *F*_c_ − *F*_o_ difference density could be observed in the region corresponding to chain *E*. Thus, we elected to leave chain *E* modelled in the final deposited structure (PDB entry 6gdt).

### 3.3. The structure of VC0615 and its relationship to other GH9 enzymes   

The structure of VC0615 is highly similar to those solved for GH9 enzymes from *P. profundum* (PBPRA0520; PDB entry 5dgr; Honda *et al.*, 2016 ▸) and *V. parahaemolyticus* (VP2484; PDB entry 3h7l; New York SGX Research Center for Structural Genomics, unpublished work), sharing 60 and 68% sequence identity, respectively. Superposition using the SSM superpose function of *CCP*4*mg* gives r.m.s.d.s of 0.66 Å over 554 residues for VC0615 and PBPRA0520, and 0.59 Å over 563 residues for VC0615 and VP2484 (comparing chain *A* of each structure).

**Figure 5:**
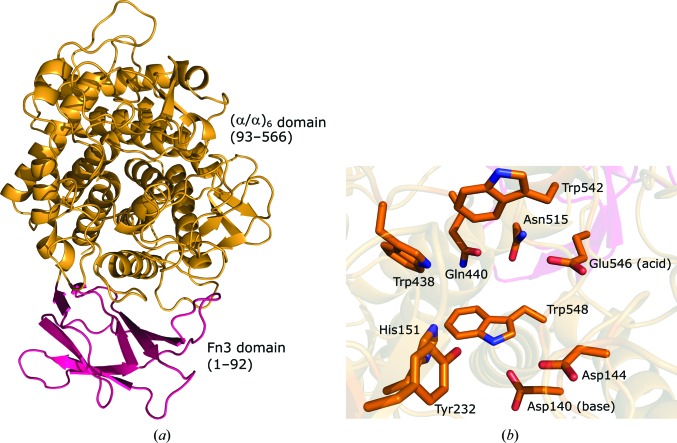
Structure of a VC0615 monomer. (*a*) Ribbon diagram of VC0615 (chain *A*), showing the Fn3 (pink) and (α/α)_6_-barrel (orange) domains. (*b*) Active-site residues of VC0615. Postulated acid and catalytic base residues are annotated.

**Figure 6:**
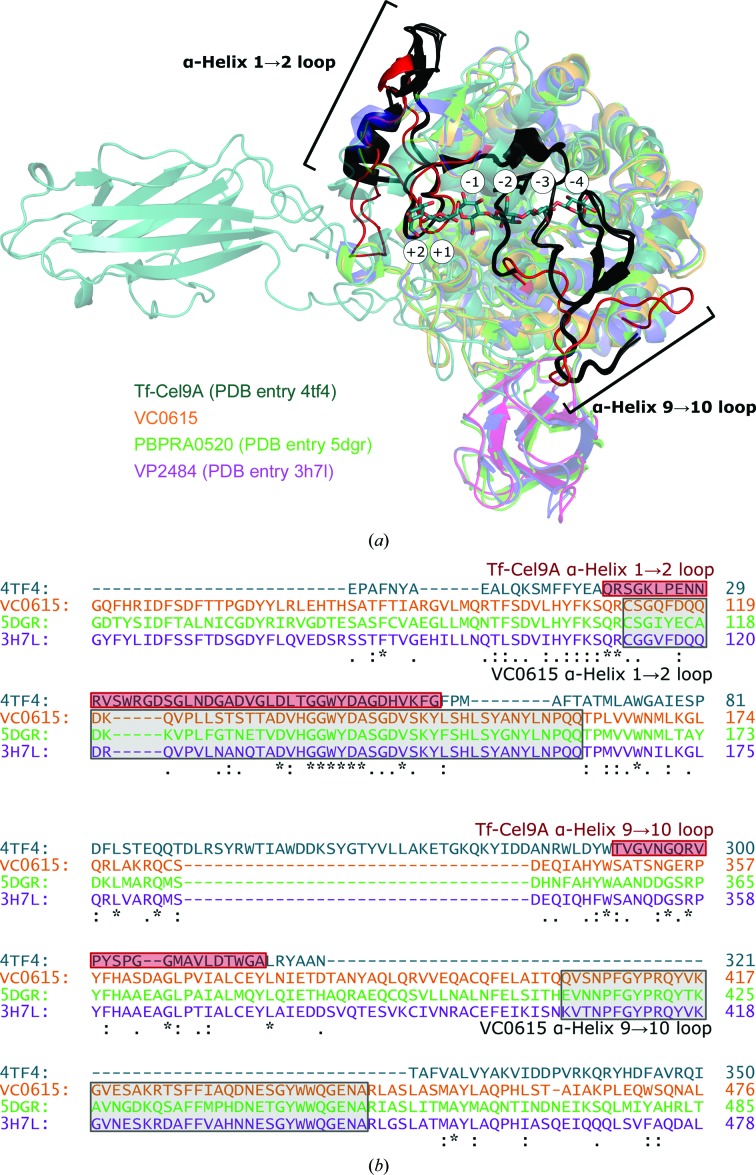
Vibrionaceae exo-acting GH9s *versus* an endo-acting GH9 enzyme. (*a*) (α/α)_6_-domain alignment of the exo-acting GH9s VC0615 (colours as in Fig. 5 ▸), PBPRA0520 (PDB entry 5drq; green) and VP2484 (PDB entry 3h7l; purple), alongside the endo-acting GH9 Tf-Cel9a from *T. fusca* (PDB entry 4tf4; teal), in complex with a cleaved cellohexaose substrate (teal sticks; white circles denote enzyme subsites). Extended loops in exo-acting GH9s from α-­helices 1 to 2 (black; 112–162 in VC0615 numbering) and α-helices 9 to 10 (black; 404–442) form steric blocks which close off the binding pockets of these enzymes. The corresponding loops in Tf-Cel9a (red; 20–63 and 292–315) are smaller and do not obstruct the substrate-binding cleft. (*b*) *Clustal Omega* (Sievers & Higgins, 2018 ▸) alignment of the VC0615, PBPRA0520, VP2484 and Tf-Cel9a sequences, with loop sequences highlighted. The large disparity in loop sizes causes suboptimal alignment by *Clustal Omega* in the α-helix 9–10 region. Colours are as in (*a*).

VC0615 protomers are comprised of two domains: an N-terminal fibronectin type III (Fn3) domain (amino acids 1–92) and a C-terminal (α/α)_6_-barrel domain (93–566) which contains the active site. The active site of VC0615 (position inferred from homology to a PBPRA0520–ligand complex; PDB entry 5dgr) is situated within a series of loops which emanate from the core helices of the (α/α)_6_-barrel (Fig. 5 ▸). Long linkers from α-helices 1–2 (residues 112–162) and α-helices 9–10 (404–442) of VC0615 (features that are well resolved at 3.17 Å resolution) contribute to the formation of a closed-off steric block towards the ‘rear’ face of the binding pocket, which restricts the active-site binding pocket to only a single sugar-binding subsite. These long loops distinguish VC0615 and related exo-acting GH9s from the endo-acting GH9s, such as Tf-Cel9A cellulase from *Thermobifida fusca*, which has much shorter loops linking its α-helices (PDB entry 4tf4; Sakon *et al.*, 1997 ▸; Fig. 6 ▸).

**Figure 7:**
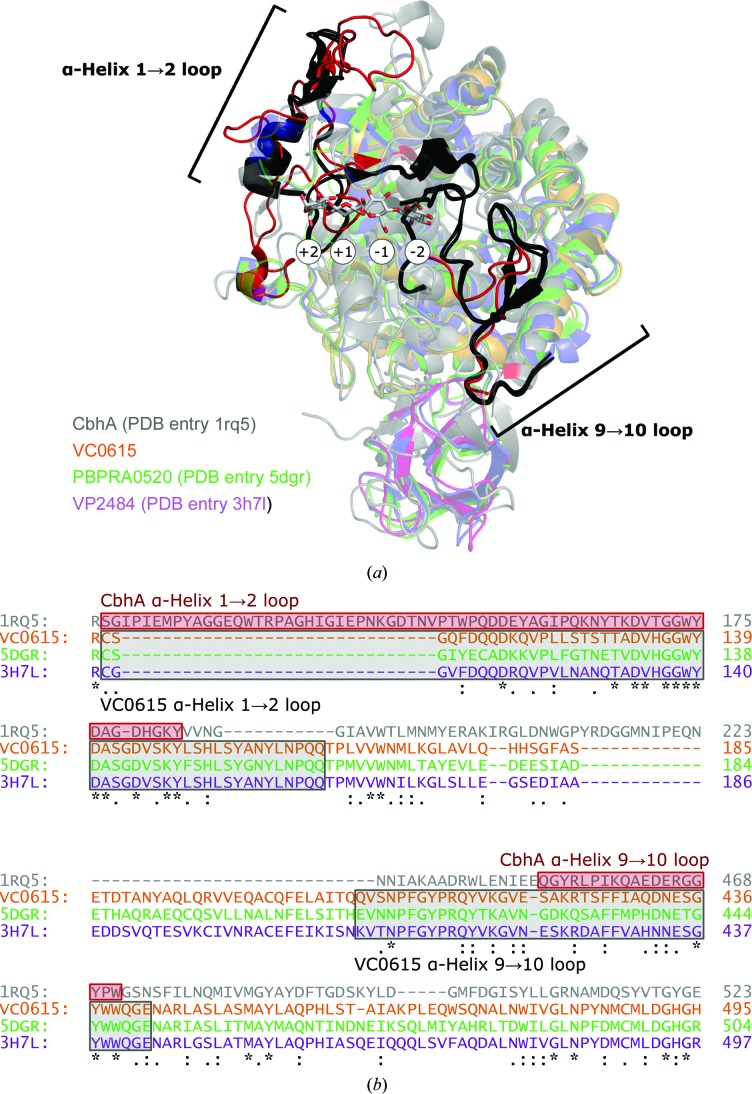
Vibrionaceae exo-acting GH9s *versus* the *C. thermocellum* cellobiohydrolase CbhA. (*a*) The exo-acting GH9s VC0615, PBPRA0520 and VP2484, alongside an CbhA E795Q mutant (PDB entry 1rq5; grey), in complex with an uncleaved cellotetraose substrate (grey sticks; white circles denote enzyme subsites). The loops in exo-acting GH9s from α-helices 1 to 2 (black; 112–162 in VC0615 numbering) and α-helices 9 to 10 (404–442) help to occlude the −2 subsite of exo-acting GH9s. The corresponding loops in CbhA (red; 117–183 and 454–471) do not occlude the −2 subsite. The α-helix 1–2 loop in exo-acting GH9s projects more ‘into’ the enzyme binding site compared with the same loop in CbhA, creating more of a steric block against –2 subsite binding. (*b*) *Clustal Omega* (Sievers & Higgins, 2018 ▸) alignment of the VC0615, PBPRA0520, VP2484 and CbhA sequences, with loops highlighted. Colours are as in (*a*).

Although not strictly an exo-acting enzyme, the GH9 cellobiohydrolase CbhA from *C. thermocellum* (PDB entry 1rq5; Schubot *et al.*, 2004 ▸) has been reported to contain a structurally closed binding pocket, albeit one which hydrolyses terminal cellobiose rather than terminal glucose units. Structural comparison of VC0615 and related exo-acting GH9s with CbhA shows that the linkers from α-helices 1 to 2 and 9 to 10 contribute more to steric blocking of the −2 subsite in the true exo-acting enzymes (Fig. 7 ▸). Notably, whilst the loop from α-helices 1 to 2 is actually longer in CbhA (117–183) compared with the exo-acting GH9s (112–162 in VC0615), the α-helix 1–2 loop in exo-acting GH9s is projected strongly ‘inwards’ towards the active site compared with CbhA, thus occluding more of the enzyme binding pocket.

The active-site residues are nearly completely conserved between VC0615, PBPRA0520 and VP2484, with the only variation being the presence of Phe231 in PBPRA0520, compared with Tyr at the equivalent position in VC0615 and VP2484 (Tyr232 in VC0615). Although we attempted to obtain a ligand complex of VC0615 by soaking crystals with cellotriose or cellotetraose, these efforts did not produce a structure with interpretable active-site electron density. Thus, we examined the active-site interactions in VC0615 *via* comparison with a PBPRA0520–glucosamine ligand complex. Based on homology to PBPRA0520, Asp140 and Glu546 of VC0615 are postulated to act as the catalytic base and acid residues of VC0615, respectively, with Asp144 also likely to be important in coordinating the incoming water nucleophile. Asp140 and Glu546 are situated 8.4 Å apart, consistent with the typical separation for catalytic residues of an inverting glycosidase (Davies & Henrissat, 1995 ▸). Surprisingly, there appears to be no structural rationale within the VC0615 (or PBPRA0520) binding pocket for the observed preference for chito-oligosaccharide over cellulosic substrates. A −1 subsite glucosamine would be expected to position its 2-amino group within hydrogen-bonding distance of Tyr148, Tyr232 and Trp220 of VC0615, none of which are obviously suited for discriminating the hydrogen-bonding pattern of an amino group over a hydroxyl and which are also incorrectly oriented (‘edge on’) to form a cation–π interaction with a charged NH_3_^+^. It is possible that the observed preference of VC0615 for chito-oligosaccharides may arise from factors outside the −1 subsite binding pocket, such as interactions with the departing +1 sugar.

## 4. Conclusion   

Here, we have reported the structure of the *V. cholerae* enzyme VC0615 at 3.17 Å resolution. The VC0615 crystal structure contains two relatively well ordered homodimers of VC0615 in each asymmetric unit (chains *A* and *D* and chains *B* and *C* in our structure), as well as a fifth highly disordered molecule of VC0615 (chain *E*) which appears to lack a dimeric partner. *PISA* analysis indicated that a third VC0615 homodimer (chains *E* and *F*) cannot be easily accommodated by the VC0615 crystal lattice owing to steric clashes which would arise between symmetry-related copies of molecules of ‘chain *F*’. Given the intrinsic dimeric nature of VC0615, it is possible that ‘chain *F*’ molecules are present within the VC0615 crystal lattice, albeit not in a sufficiently ordered fashion to contribute to X-ray diffraction. Difficulty in packing molecules of ‘chain *F*’ within the VC0615 crystal lattice provides a clear explanation for the high degree of disorder observed in molecules of chain *E*, and may also be a substantial contributing factor to the poor diffraction quality of VC0615 crystals in general.

VC0615 forms part of an exo-acting glucosidase/glucos­aminidase subgroup within the CAZy GH9 family, alongside previously reported structures of PRPBA0520 and VP2484. Structural comparison of the exo­-acting GH9s with both an endo-acting GH9 cellulase and a GH9 cellobiohydrolase illustrate that the loop topologies around the catalytic (α/α)_6_ domain are key for delineating substrate accessibility for all GH9 enzymes. In particular, loops from α-helices 1 to 2 and α-­helices 9 to 10 cause steric blocks within the (α/α)_6_ domain of exo-acting GH9s, which restrict substrate binding beyond the −1 subsite.

Some key questions remain regarding the interaction of exo-acting GH9s such as VC0615 with their substrates. In particular, no obvious basis for the discrimination of the −1 subsite for GlcN over Glc has yet been discerned in the structures of VC0615, PBPRA0520 or VP2484, implying the possibility of GlcN discrimination at the departing +1 subsite of the enzyme. This is supported by the observation by Honda and coworkers that whilst (GlcN)_2_ is hydrolysed by PBPRA0520 with a *k*_cat_/*K*_m_ almost 15 times greater than for (Glc)_2_, the *k*_cat_/*K*_m_ for the hydrolysis of the aryl substrate pNP-GlcN is only approximately threefold greater than that for pNP-Glc (Honda *et al.*, 2011 ▸). The hypothesis by Hunt and coworkers that VC0615 acts on a GlcN-GlcN-O6-P substrate *in vivo* (Hunt *et al.*, 2008 ▸) also remains to be tested, especially with regard to the potential binding contributions made by an O6 phosphate at the +1 enzyme subsite. Improved structural and biochemical understanding of exo-­acting GH9 enzymes will help to shed light on these questions, as well as broader questions regarding the molecular basis of chitin utilization by the Vibrionaceae.

## Figures and Tables

**Table 1:** Macromolecule-production information

Source organism	*V. cholerae*
DNA source	Synthesized cDNA
Forward primer	T7-fwd, TAATACGACTCACTATAGGG (for sequencing only)
Reverse primer	pET-RP, CTAGTTATTGCTCAGCGG (for sequencing only)
Cloning vector	pET-21a
Expression vector	pET-21a
Expression host	*E. coli* BL21 Gold (DE3)
Complete amino-acid sequence of the construct produced	MLLLTNHIGYETQGPKQAVLLCGQTQLMDDCVLLVCARSHQTVAKLAIEWHGKVDNWHQGQFHRIDFSDFTTPGDYYLRLEHTHSATFTIARGVLMQRTFSDVLHYFKSQRCSGQFDQQDKQVPLLSTSTTADVHGGWYDASGDVSKYLSHLSYANYLNPQQTPLVVWNMLKGLAVLQHHSGFASFSRTRLKDEALFGADFLRRMQNSEGFFYMTVFDKWSKDTKQREICAYATQQGHKSDDYQAGFRQGGGMAIAALAAAARLDTHGEFTQADYLQAAENGYWHLKEHNLAYLNDGVENIIDEYCALLACCELYRTTENDQYLAQAREWAQRLAKRQCSDEQIAHYWSATSNGERPYFHASDAGLPVIALCEYLNIETDTANYAQLQRVVEQACQFELAITQQVSNPFGYPRQYVKGVESAKRTSFFIAQDNESGYWWQGENARLASLASMAYLAQPHLSTAIAKPLEQWSQNALNWIVGLNPYNMCMLDGHGHNNPDYLPHLGFFNAKGGVCNGITAGFDDPRDIAFNPAGQKDDMLQNWRWGEQWIPHGAWYLLAIISQFAHFTAHGEENQLEHHHHHH

**Table 2:** Crystallization

Method	Sitting-drop vapour diffusion for initial crystallization trials; hanging-drop vapour diffusion for optimization trials
Plate type	MRC 96-well 2-drop plates for initial trials; 24-well sterile tissue-culture plates for optimized conditions
Temperature (K)	293
Protein concentration (mg ml^−1^)	67.5
Buffer composition of protein solution	20 m*M* HEPES pH 7.4, 100 m*M* NaCl, 1 m*M* DTT
Composition of reservoir solution	100 m*M* Tris pH 7.5, 5% PGA-LM, 8% PEG 20K (final optimized conditions)
Volume and ratio of drop	120 nl protein + 120 nl reservoir solution (initial trials); 1 µl protein + 1 µl reservoir solution (final optimized conditions)
Volume of reservoir	54 µl (initial trials); 500 µl (final optimized conditions)

**Table 3:** Data collection and processing Values in parentheses are for the outer shell.

Diffraction source	I03, DLS
Wavelength (Å)	0.9762
Temperature (K)	100
Detector	PILATUS3 6M
Crystal-to-detector distance (mm)	557.15
Rotation range per image (°)	0.10
Total rotation range (°)	220
Exposure time per image (s)	0.040
Space group	*P*3_2_21
*a*, *b*, *c* (Å)	234.90, 234.90, 129.42
α, β, γ (°)	90, 90, 120
Mosaicity (°)	0.26
Resolution range (Å)	47.33–3.17 (3.24–3.17)
Total No. of reflections	801107
No. of unique reflections	69816
Completeness (%)	100 (100)
Multiplicity	11.5 (10.8)
〈*I*/σ(*I*)〉	8.4 (0.9); <2 at ∼3.5 Å
Half-set correlation (CC_1/2_)	1.0 (0.32)
*R*_p.i.m._	0.08 (0.90)
Overall *B* factor from Wilson plot (Å^2^)	74

**Table 4:** Structure solution and refinement Values in parentheses are for the outer shell.

PDB code	6gdt
Resolution range (Å)	47.33–3.17 (3.24–3.17)
Completeness (%)	100 (100)
No. of reflections, working set	66351
No. of reflections, test set	3411
Final *R*_cryst_	0.23
Final *R*_free_	0.28
Cruickshank DPI	0.52
No. of non-H atoms	
Protein	22634
Chains *A*–*D*	18128
Chain *E*	4506
Ligand	4
Solvent	71
R.m.s. deviations	
Bonds (Å)	0.007
Angles (°)	1.13
Average *B* factors (Å^2^)	
Protein	107
Chains *A*–*D*	89.6
Chain *E*	175.3
Ligand	77
Water	55
Ramachandran plot	
Most favoured (%)	93.9
Allowed (%)	4.1
